# TMU-Net: A Transformer-Based Multimodal Framework with Uncertainty Quantification for Driver Fatigue Detection

**DOI:** 10.3390/s25175364

**Published:** 2025-08-29

**Authors:** Yaxin Zhang, Xuegang Xu, Yuetao Du, Ningchao Zhang

**Affiliations:** School of Electronic Information Engineering, Xi’an Technological University, Xi’an 710021, China; zhangyaxin@st.xatu.edu.cn (Y.Z.); xuxuegang@st.xatu.edu.cn (X.X.); zhangningchao@xatu.edu.cn (N.Z.)

**Keywords:** driver fatigue detection, multimodal fusion, electroencephalogram (EEG), electrooculogram (EOG), uncertainty quantification

## Abstract

**Driving fatigued** is a prevalent issue frequently contributing to traffic accidents, prompting the development of automated fatigue detection methods based on various data sources, particularly reliable physiological signals. However, challenges in accuracy, robustness, and practicality persist, especially for cross-subject detection. Multimodal data fusion can enhance the effective estimation of driver fatigue. In this work, we leverage the advantages of multimodal signals to propose a novel Multimodal Attention Network (TMU-Net) for driver fatigue detection, achieving precise fatigue assessment by integrating electroencephalogram (EEG) and electrooculogram (EOG) signals. The core innovation of TMU-Net lies in its unimodal feature extraction module, which combines causal convolution, ConvSparseAttention, and Transformer encoders to effectively capture spatiotemporal features, and a multimodal fusion module that employs cross-modal attention and uncertainty-weighted gating to dynamically integrate complementary information. By incorporating uncertainty quantification, TMU-Net significantly enhances robustness to noise and individual variability. Experimental validation on the SEED-VIG dataset demonstrates TMU-Net’s superior performance stability across 23 subjects in cross-subject testing, effectively leveraging the complementary strengths of EEG (2 Hz full-band and five-band features) and EOG signals for high-precision fatigue detection. Furthermore, attention heatmap visualization reveals the dynamic interaction mechanisms between EEG and EOG signals, confirming the physiological rationality of TMU-Net’s feature fusion strategy. Practical challenges and future research directions for fatigue detection methods are also discussed.

## 1. Introduction

Fatigue driving poses a significant challenge to global road safety. According to statistics from the World Health Organization, approximately 12–20% of road traffic accidents are directly attributed to driver fatigue [1]. As is well-documented, fatigue impairs perception, memory, decision-making, and judgment, thereby increasing the risk of traffic accidents [2,3]. A recent report by the National Highway Traffic Safety Administration (NHTSA) revealed that in the United States alone, fatigue-related driving accidents exceed 100,000 annually, resulting in severe casualties and substantial economic losses [4]. This critical situation has spurred continuous efforts in both academia and industry to explore more effective driver fatigue detection technologies.

Recent studies have primarily pursued three technical approaches for driver fatigue detection. (1) Vehicle motion-based methods analyze vehicular dynamic parameters such as steering wheel angle, speed, and acceleration. Research indicates that these methods achieve high detection accuracy in structured road environments [5]; however, their performance is significantly influenced by road complexity, with accuracy potentially declining markedly in urban settings [6]. (2) Driver behavior-based methods employ computer vision to monitor facial features (e.g., eye closure duration, yawning frequency) [7]. The hybrid LSTM-CNN model developed by Quddus et al. [8] attained 89.7% accuracy under standardized test conditions. Nevertheless, such methods face limitations in low-light conditions or when drivers wear glasses [9], and struggle to distinguish fatigue from transient eye closures [10]. (3) Physiological signal-based methods leverage biomarkers, among which PERCLOS (Percentage of Eyelid Closure Over the Pupil) is widely regarded as the gold standard for fatigue assessment [11]. Initially proposed by Dinges et al. in NHTSA-sponsored research [11], PERCLOS was later validated by Wierwille et al. through large-scale on-road experiments, demonstrating strong correlation with actual driver fatigue states [12].

Further studies demonstrate that when PERCLOS values exceed 0.15, drivers exhibit a significantly elevated risk of accidents [13]. However, computer vision-based PERCLOS detection faces substantial challenges in real-world driving conditions. Bergasa et al. [13] reported that detection accuracy may decrease by over 40% in low-light environments (e.g., nighttime or tunnels). Ji et al. [14] found that head rotations exceeding 15° markedly increase measurement errors, while Garcia et al. [15] noted that obstructions like eyeglasses or sunglasses can lead to a 35% failure rate in PERCLOS detection. Additionally, interethnic variations in palpebral fissure height (2–3 mm) further compromise measurement consistency [15].

These limitations have driven researchers toward more reliable direct physiological signal detection methods. Multimodal physiological signal fusion approaches, which integrate information from diverse modalities (e.g., electroencephalography (EEG) for brain activity and electrooculography (EOG) for eye movements), effectively overcome the constraints of single-signal systems [16]. Zheng and Lu [17] demonstrated that such multimodal methods offer distinct advantages over traditional PERCLOS detection, including the following: Robustness to environmental interference; earlier fatigue state detection; and direct measurement of neurological activity.

Current multimodal frameworks for driver fatigue detection predominantly adopt three fusion strategies: (1) Early fusion methods, exemplified by Zheng et al.’s EEG-EOG feature-level concatenation model [18], demonstrate computational efficiency but suffer from information redundancy and feature interference, with studies showing 15–20% accuracy degradation when handling modalities with significant sampling rate disparities [19]; (2) late fusion approaches, like Luo et al.’s decision-level system [20], enable modular asynchronous processing but require stringent temporal alignment and may disregard cross-modal correlations; and (3) hybrid methods, such as Cheng et al.’s VigilanceNet [21], achieve optimal laboratory performance, yet their computational complexity hinders real-time implementation in actual driving scenarios. Recent advances in multimodal information processing have provided insights for optimizing fusion strategies. For instance, Cai et al. [22] proposed a visual grounding-based reinforcement-learning framework that enhances model robustness in dynamic environments through object-level feature integration. This approach offers valuable reference for the multimodal fusion of EEG and EOG signals, particularly in addressing signal interference issues in complex driving scenarios.

Through a systematic analysis of the existing literature, we identified several critical limitations in current research. (i) Absence of dynamic reliability assessment—signal quality fluctuates with motion artifacts (EEG contamination in 23–41% of recordings [16]) and environmental noise (EOG variance during head movements [23]), while current static-weighting schemes fail to adapt; (ii) inadequate uncertainty quantification—despite evidence that probabilistic modeling enhances noise robustness by 18–22% [24], most systems neglect this capability; (iii) limited cross-subject generalization—model performance typically degrades by 30–40% for new subjects due to physiological variability, where adversarial domain adaptation [25] reduces this gap by 12% at the expense of tripled computational overhead.

This study aims to develop a robust and accurate driver fatigue detection framework (TMU-Net) to address the limitations of existing methods. To this end, we propose key design choices involving multimodal fusion (EEG + EOG) and the simultaneous use of 2 Hz full-band and five-band EEG features. Single physiological modalities are susceptible to noise and provide limited information. Electroencephalogram (EEG) signals directly reflect changes in central neural activity, serving as an early internal indicator of fatigue, but are prone to contamination from electromyographic and motion artifacts. In contrast, electrooculogram (EOG) signals capture prominent external behavioral manifestations of fatigue, exhibit high correlation with PERCLOS, and offer greater stability, though their reliability may be affected by visual obstructions or lighting variations. Therefore, fusing EEG and EOG leverages the complementary strengths of the “internal and early” nature of EEG and the “external and direct” nature of EOG, enhancing system robustness across diverse scenarios. Concurrently, 2 Hz full-band features capture subtle, frequency-specific variations in brain activity, providing a rich data foundation and improving the model’s sensitivity to minor fatigue changes. Conversely, the classical five-band features are linked to established physiological mechanisms, offering strong interpretability and smoothing noise to some extent through spectral consolidation, thus yielding more stable feature representations. By integrating both feature sets, the model benefits from the sensitivity of high-resolution data and the robustness and interpretability of standard frequency bands.

The main contributions of this paper are summarized as follows:

1. We propose TMU-Net, a novel framework that for the first time integrates EEG signals (both 2 Hz full-band and five-band features) with EOG signals, incorporating uncertainty modeling to enhance the accuracy and robustness of driver fatigue detection.
2. We design a unimodal feature extraction module that employs causal convolution, ConvSparseAttention, and Transformer encoders to capture spatiotemporal features, while integrating uncertainty modeling to improve prediction reliability.
3. We dynamically integrate features through a weighted gating mechanism, thereby achieving adaptive optimization of fatigue detection performance.

## 2. Methodology

**Figure 1:**
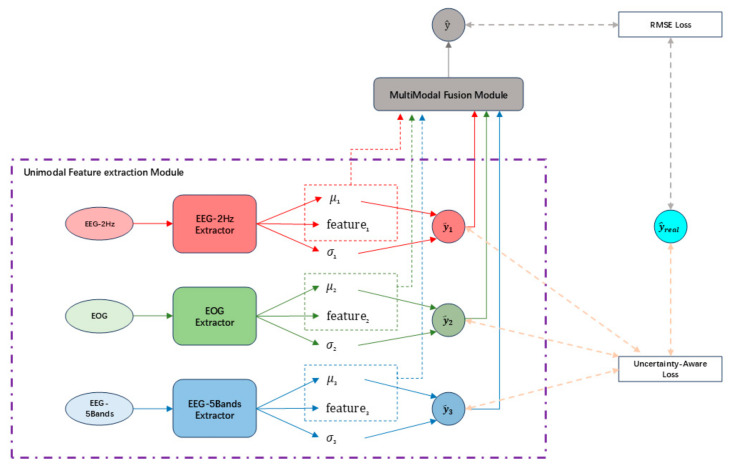
The overall architecture of TMU-Net.

In this section, we present our model, TMU-Net. As illustrated in Figure 1, the model architecture primarily consists of two core modules: the unimodal feature extraction module and the multimodal fusion module. Raw EEG and EOG signals are first processed independently by the unimodal module, which extracts spatiotemporal features and estimates uncertainty for each modality. These features, along with their uncertainty estimates, are then fed into the multimodal fusion module, where cross-modal attention and uncertainty-weighted gating mechanisms dynamically integrate the information to produce the final fatigue prediction. These two modules will be described in detail in Section 2.1 and Section 2.2.

### 2.1. Unimodal Feature Extraction Module

**Figure 2:**
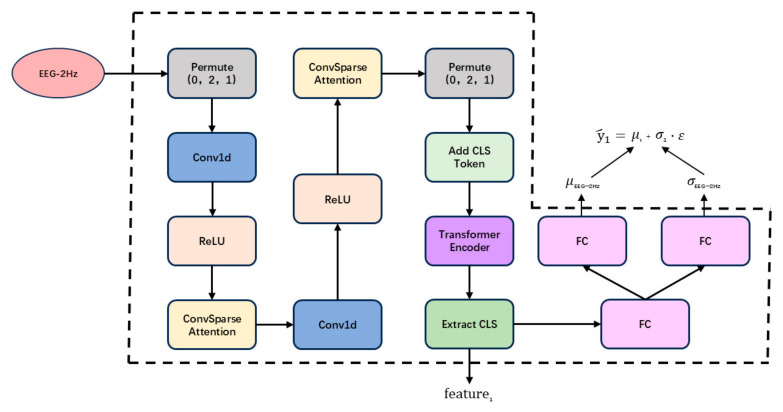
Structural Diagram of 2 Hz EEG Feature Extractor.

The unimodal feature extraction module is a core component of TMU-Net, designed to extract time-frequency features from each modality while quantifying prediction reliability through uncertainty modeling. This is achieved by integrating causal convolution, a convolutional sparse attention mechanism (ConvSparseAttention), and a Transformer encoder. Taking the EEG-2Hz modality as an example, the detailed architecture of the feature extractor is illustrated in Figure 2. The feature extractors for other modalities (e.g., EOG and EEG-5Bands) follow a similar structure, with only parameter configurations differing.

Let the input data of EEG-2Hz be denoted as $x\in R^{B\;\times \;S\;\times \;D}$, where B represents the batch size, S denotes the time series length, and D indicates the feature dimension. The input data undergoes a dimension permutation, transforming from $R^{B\;\times \;S\;\times \;D}$ to $R^{B\;\times \;D\;\times \;S}$, to accommodate the 1D convolution operation.

The input data undergoes local feature extraction through a two-layer 1D causal convolutional neural network (CNN). Each convolutional layer is followed by a ReLU activation function, and causal padding is applied to preserve the temporal causality of the sequence. After each convolutional layer, a ConvSparseAttention module is introduced to enhance the spatial and temporal representation capabilities of the features.

Compress the input channels into a single channel using a one-dimensional convolution (kernel_size = 1) to generate a feature map of shape [B×1×S], which is then repeatedly expanded to $\left[B\times C_{out}\times S\right]$.

Apply depthwise convolution ($groups=C_{out}$) followed by pointwise convolution to enhance temporal interactions while preserving channel independence, with ReLU activation introducing nonlinearity.

The features are transposed to $\left[B\times S\times C_{out}\right]$ dimensions, followed by computation of attention weights through a multi-head attention mechanism. These weights are normalized via Softmax to generate attention-weighted features.

Features are extracted through the other convolutional pathway consisting of 1D convolution, batch normalization, and ReLU activation. These features undergo element-wise multiplication with the attention-weighted features, followed by integration with the original input features via residual connection.

Subsequently, a Transformer encoder processes the sequence, with a CLS token appended to aggregate global information. The CLS token output is split into two branches:

One branch generates the mean prediction via linear transformation:

(1) $$\mu_{1}=W_{\mu_{1}}x_{cls}+b_{\mu_{1}}$$

The other branch produces the logarithm of the variance through a linear transformation, followed by an exponential function to ensure non-negativity:

(2) $${\sigma_{1}}^{2}=e^{\left(W_{\sigma_{1}}x_{cls}+b_{\sigma_{1}}\right)}$$

Under the Gaussian distribution assumption, the predicted value is generated as follows:

(3) $${\hat{y}}_{1}=\mu_{1}+\sqrt{{\sigma_{1}}^{2}}\cdot \varepsilon$$

where ε~N(0,1) is random noise introduced during training, following a standard normal distribution.

The unimodal feature extraction module of TMU-Net extracts time-frequency features from EEG and EOG signals while quantifying prediction reliability via uncertainty modeling. Causal convolution ensures temporal causality for real-time driver fatigue detection. ConvSparseAttention merges CNNs’ local pattern capture with attention mechanisms’ long-range dependency modeling for efficient feature extraction. The Transformer encoder captures global context. Uncertainty estimation enables dynamic weighting in the fusion module, enhancing robustness over static averaging.

### 2.2. Multimodal Feature Fusion Module

**Figure 3:**
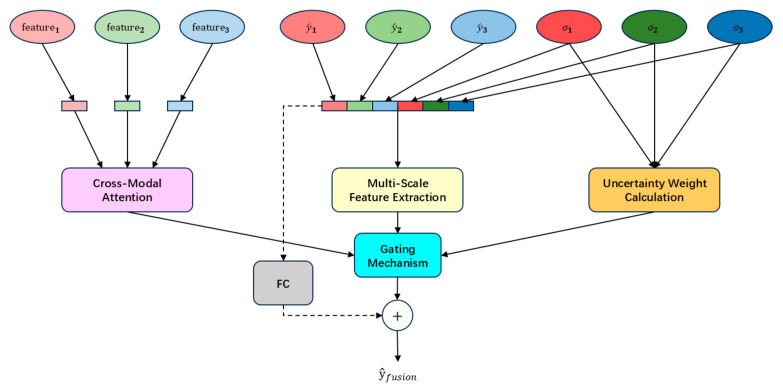
Structural Diagram of MultiModalFusion.

The multimodal fusion module integrates the predicted values, feature representations, and uncertainty estimates derived from EEG-2Hz, EOG, and EEG-5Bands modalities to generate the final fused prediction ${\hat{y}}_{fusion}\in R^{B\;\times \;1}$. This module achieves dynamic fusion through multi-scale feature extraction, cross-modal attention mechanisms, gating operations, and residual connections. The detailed structure of the module is illustrated in Figure 3.

The multimodal fusion module receives the outputs from the unimodal feature extractors, including the following:

The unimodal prediction values ${\hat{y}}_{1},\;{\hat{y}}_{2},\;{\hat{y}}_{3}$, corresponding to the predictions from EEG-2Hz, EOG, and EEG-5Bands modalities, respectively.

The predictive uncertainty variances ${\sigma_{1}}^{2},{\sigma_{2}}^{2},{\sigma_{3}}^{2}$, representing the uncertainty associated with each modality’s prediction.

The feature representations extracted from the CLS token outputs, denoted as ${feature}_{1},\;{feature}_{2},\;{feature}_{3}$.

To model the interactions between modalities, the CLS token features from each modality to a unified dimensional space of size $d_{model}$ through a linear transformation. The projected features are then stacked to form a tensor ($features\in R^{B\;\times \;3\;\times \;d_{model}}$).

**Figure 4:**
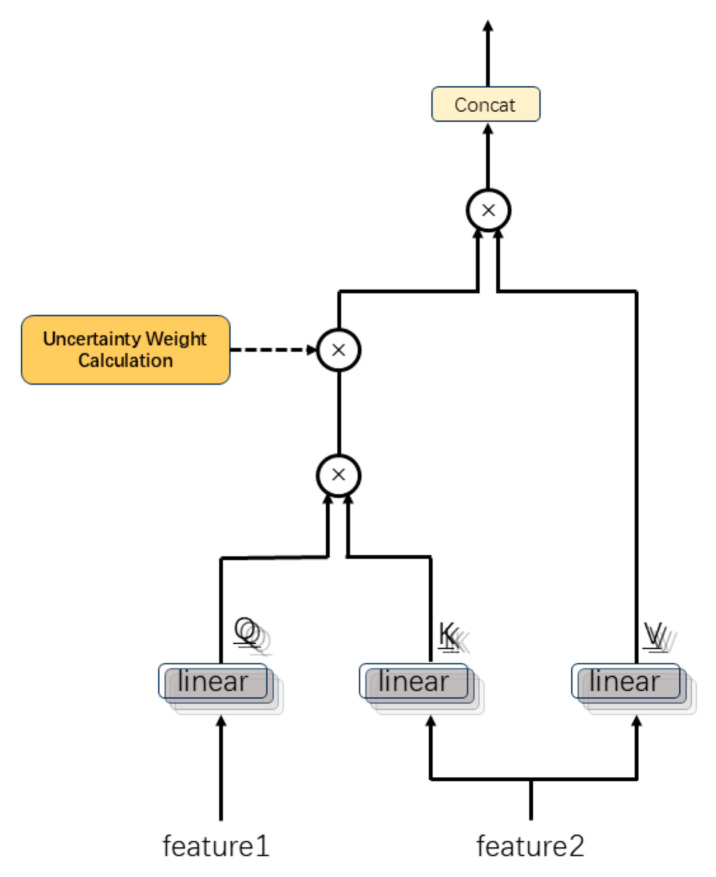
Architecture of multi-head attention between two channels.

As shown in the Figure 4, for each modality-specific feature, query (Q), key (K), and value (V) vectors are generated as follows:

(4) $$Q=features{\cdot W}_{Q}+b_{Q},\;K=features{\cdot W}_{K}+b_{K},\;V=features{\cdot W}_{V}+b_{V}$$

where $W_{Q},W_{K},W_{V}\in R^{d_{model}\;\times \;d_{model}}$ and $b_{Q},b_{K},b_{V}\in R^{model}$, and the resulting tensors are $Q,K,V\in R^{B\;\times \;3\;\times \;d_{model}}$.

The Q,K,V tensors are split into $n_{h}=4$ attention heads, with each head having dimensionality$\;d_{h}=\frac{d_{model}}{n_{h}}$, Accordingly, the head-specific tensors are denoted as follows: $Q_{h},K_{h},V_{h}\in R^{B\;\times \;n_{h}\;\times \;3\;\times \;d_{h}}$.

For each attention head, the cross-modal attention score is computed as follows:

(5) $$S_{h}=\frac{Q_{h}\cdot K_{h}^{T}}{\sqrt{d_{h}}}$$

where $\sqrt{d_{h}}$ serves as a scaling factor to mitigate the numerical instability of high-dimensional dot-product operations.

The uncertainty of each modality is represented by its variance. Precision is defined as the inverse of the variance:

(6) $$\pi_{i}=\frac{1}{\sigma_{i}^{2}+\varepsilon },\;\;i=1,2,3$$

where $\varepsilon =10^{-8}$ is a constant added to avoid division by zero, $\pi_{i}\in R^{B\;\times \;1}$ serves as the precision of modality *i*, with higher values indicating lower uncertainty.

The relative confidence weights are computed for each modality based on their precision and normalized as follows:

(7) $$w_{i}=\frac{\pi_{i}}{\sum_{j}^{3}\pi_{j}},\;\;i=1,2,3$$

where the normalization ensures $\sum_{i}^{3}w_{i}=1$, enhancing both interpretability and stability of the weights, with $w_{i}\in R^{B\;\times \;1}$.

An initial confidence weight vector is constructed as follows:

(8) $$W_{raw}=\left[w_{1},w_{2},w_{3}\right]\in R^{B\;\times \;3}$$

$W_{raw}$ is mapped to the model dimension $d_{model}$ through a linear projection layer.

To align with the dimensionality requirements of the multi-head attention mechanism, we perform expansion and reshaping operations:

(9) $$W^{\prime }=reshape\left(repeat\left(W_{raw},n_{h}\right)\right)\in R^{B\;\times \;n_{h}\;\times \;3\;\times \;d_{h}}$$

The projected weights are adapted for multi-head attention through dimensional transformations, modulating attention scores to prioritize high-confidence modalities:

(10) $$S_{h}^{\prime }=S_{h}\cdot softmax\left(W_{proj}\cdot \beta ,dim=-1\right)$$

where β=0.5 serves as a scaling factor to control the intensity of confidence weights, and the softmax operation normalizes along the modality dimension.

The attention output is generated as follows:

(11) $$O_{h}=S_{h}^{\prime }V_{h}$$

where $O_{h}\in R^{B\;\times \;n_{h}\;\times \;3\;\times \;d_{h}}$ incorporates both cross-modal interaction information and uncertainty weights:

(12) $$O=Concat\left(O_{1},\ldots ,O_{n_{h}}\right)W_{O}+b_{O}$$

with learnable parameters $W_{O}\in R^{n_{h}\;\times \;d_{h}\;\times \;d_{model}}$ and ${\;b}_{O}\in R^{d_{model}}$ to restore the model dimension $d_{model}$.

Then, we perform modality-wise averaging to produce the fused feature representation:

(13) $${feature}_{fused}=\frac{1}{3}\sum_{i=1}^{3}O\left[:,i,:\right]$$

The resulting ${feature}_{fused}\in R^{B\;\times \;d_{model}}$ integrates feature representations from all three modalities (EEG-2Hz, EOG, EEG-5Bands), combining cross-modal interactions with uncertainty-weighted information, which serves as input for subsequent gating mechanisms.

The multimodal fusion module simultaneously receives both the predicted values ${\hat{y}}_{1},{\hat{y}}_{2},{\hat{y}}_{3}$ from single-modality feature extractors and their uncertainty variances ${\sigma_{1}}^{2},{\sigma_{2}}^{2},{\sigma_{3}}^{2}$, concatenating to form the input tensor:$x_{fusion}=\left[{\hat{y}}_{1},{\hat{y}}_{2},{\hat{y}}_{3},{\sigma_{1}}^{2},{\sigma_{2}}^{2},{\sigma_{3}}^{2}\right]\in R^{B\;\times \;6}.$

This tensor is further processed by a multi-scale feature extractor to capture the complex relationships between the predicted values and their associated uncertainties.

**Figure 5:**
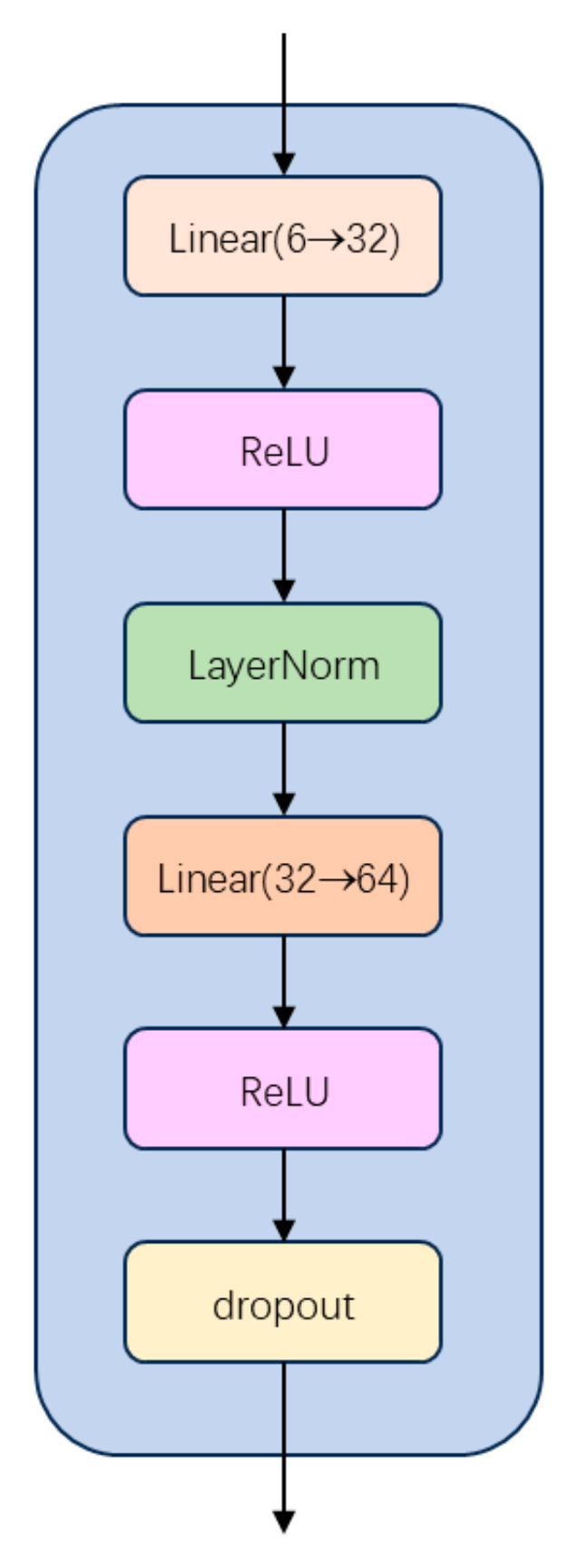
Architecture of Multi-Scale Feature Extraction.

As illustrated in Figure 5, the multi-scale feature extractor first projects the dimensionality from 6 to 32. LayerNorm is then applied to stabilize training and accelerate convergence. A second linear layer further projects the dimensionality to 64, followed by dropout with probability 0.3 to prevent overfitting:

This module extracts deep features of $x_{fusion}$ through multi-scale transformations, providing rich representations $x_{fusion_{enhanced}}$ for subsequent gated fusion.

**Figure 6:**
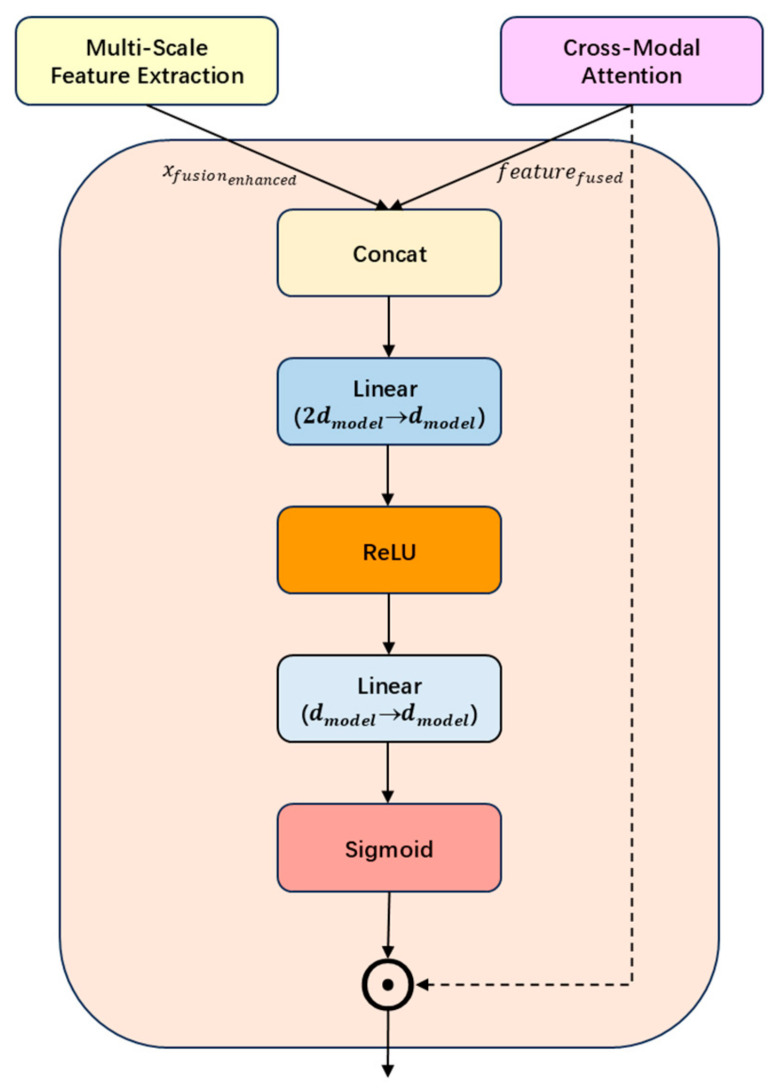
Illustration of Gating Mechanism.

As illustrated in Figure 6, the gating mechanism dynamically adjusts the contributions of cross-modal attention outputs and multi-scale features. The input is formed by concatenating the two representations: $G_{in}=[x_{fusion_{enhanced}},\;{feature}_{fused}]\in R^{B\;\times \;2d_{model}}$

The gating weights are generated via a fully connected network, which first projects the dimensionality from $2d_{model}$ to $d_{model}$ and then applies a Sigmoid activation to produce normalized weights:

(14) $$G=Sigmoid\left(W_{G}^{\prime }(ReLU\left(W_{G}G_{in}+b_{G}\right))+b_{G}^{\prime }\right)$$

With $W_{G}\in R^{2d_{model}\;\times \;d_{model}},b_{G}\in R^{d_{model}},{\;W}_{G}^{\prime }\in R^{d_{model}\;\times \;d_{model}},b_{G}^{\prime }\in R^{d_{model}}$.

The final gated output is fused via element-wise multiplication:

(15) $$G_{final}=G⊙{feature}_{fused}$$

Here, $G_{final}\in R^{B\;\times \;d_{model}}$ further dynamically modulates the weights of the fused features through the gating mechanism, ensuring that higher-confidence modalities dominate the final representation.

The final prediction is generated by integrating two branches, which combine the fused features and the original input information: the fusion connection branch and the residual connection branch.

The fusion connection branch processes the output from the gating mechanism, denoted as $G_{final}$.

First, the first linear layer maps the input dimension from d_model to 32 dimensions, followed by a ReLU activation function and a dropout layer with a dropout rate of 0.3 to obtain$\;h_{1}$.

The second linear layer then maps the dimension from 32 to a single dimension, generating the fusion prediction ${\hat{y}}_{fused}$

${\hat{y}}_{fused}\in R^{B\;\times \;1}$ denotes the prediction result obtained via the integration of cross-modal attention and gating mechanisms, which synthesizes features and uncertainty information from EEG-2Hz, EOG, and EEG-5Bands modalities.

This branch processes the initial input $x_{fusion}$ of the multimodal fusion module, where $x_{fusion}=[{\hat{y}}_{1},{\hat{y}}_{2},{\hat{y}}_{3},{\sigma_{1}}^{2},{\sigma_{2}}^{2},{\sigma_{3}}^{2}]\in R^{B\;\times \;6}$ contains the single-modality prediction values and uncertainty variances.

The first linear layer maps the input dimension from 6 to 32, followed by a ReLU activation function to obtain $h_{r}$. A normalization layer applies LayerNorm to stabilize training and enhance numerical stability.

The second linear layer maps the dimension from 32 to a single dimension, generating the residual prediction:

(16) $${\hat{y}}_{residual}=W_{r2}h_{r}+b_{r2}$$

where $W_{r2}\in R^{32\;\times \;1},\;b_{r2}\in R^{1}$ are learnable parameters, and ${\hat{y}}_{residual}\in R^{B\;\times \;1}\;$represents the direct contribution based on single-modality predictions and variances, retaining the original input information.

The outputs of the fusion connection branch and the residual connection branch are combined through addition to generate the final prediction:

(17) $${\hat{y}}_{fusion}={\hat{y}}_{fused}+{\hat{y}}_{residual}$$

The residual connection fuses the predictions from both branches by addition, preventing information loss and enhancing the model’s ability to model complex modality relationships.

The multimodal fusion module achieves dynamic fusion through multi-scale feature extraction, cross-modal attention, gating operations, and residual connections. Multi-scale feature extraction captures complex relationships between predictions and uncertainties, stabilizing training. Cross-modal attention models complementary inter-modal interactions, prioritizing high-confidence modalities. Gating operations dynamically adjust feature contributions, enhancing adaptability. Residual connections integrate unimodal and cross-modal information, preventing information loss. These designs ensure TMU-Net’s robust, high-accuracy fatigue prediction in driving scenarios.

### 2.3. Design of the Model Loss Function

To ensure that TMU-Net effectively integrates multimodal signals and enhances the accuracy and robustness of fatigue detection, we have designed a comprehensive loss function that combines the single-modality uncertainty loss, multimodal fusion loss, and auxiliary branch loss, aiming to optimize the feature extraction and dynamic fusion processes.

**Single-Modality Uncertainty Loss:** For each modality (EEG-2Hz, EOG, and EEG-5Bands), we use the negative log-likelihood (NLL) loss, combining the predicted mean and variance, while introducing a regularization term to prevent excessively large uncertainty:

(18) $$L_{unimodal,i}=\frac{1}{B}\sum_{b=1}^{B}\left[\frac{1}{2}\log\left(\sigma_{b,i}^{2}\right)+\frac{1}{2}\frac{{\left(\mu_{b,i}-y_{b}\right)}^{2}}{\sigma_{b,i}^{2}}+\lambda_{reg}\sigma_{b,i}^{2}\right]$$

where i∈{1,2,3} represents the modality, ${\hat{y}}_{b,i}$ is the predicted value for modality i, $\sigma_{b,i}^{2}$ is the uncertainty variance, $y_{b}$ is the true label, and $\lambda_{reg}=0.001$ is the regularization coefficient.

The total single-modality loss is the weighted sum of the three modalities, with weights dynamically computed based on uncertainty:

(19) $$L_{unimodal}=\sum_{i=1}^{3}w_{i}L_{unimodal,i}\;,\;\;w_{i}=\frac{\pi_{i}}{\sum_{j=1}^{3}\pi_{j}}\;,\;\;\pi_{i}=\frac{1}{\sigma_{b,i}^{2}+\varepsilon^{\prime }}\;,\;\;\;\varepsilon =10^{-8}$$

The dynamic weight $w_{i}$ ensures that modalities with higher confidence (lower variance) contribute more to the loss.

**Multimodal Fusion Loss:** The final fused prediction ${\hat{y}}_{fusion}$ is supervised using mean squared error (MSE):

(20) $$L_{fusion}=\frac{1}{B}\sum_{b=1}^{B}{\left({\hat{y}}_{b,fusion}-y_{b}\right)}^{2}$$

**Auxiliary Branch Loss:** The fusion branch (${\hat{y}}_{fused}$) and the residual branch (${\hat{y}}_{residual}$), are supervised separately to ensure their effective collaboration:

(21) $$L_{aux}=\frac{1}{B}\sum_{b=1}^{B}\left({\left({\hat{y}}_{b,fused}-y_{b}\right)}^{2}\right.+\left.{\left({\hat{y}}_{b,residual}-y_{b}\right)}^{2}\right)$$

**Total Loss Function:** The total loss function is defined as a weighted sum of three components:

(22) $$L_{total}=\alpha L_{unimodal}+{\beta L}_{fusion}+\gamma L_{aux}$$

Here, α, β, and γ are hyperparameters set to 1.0, 1.0, and 0.5, respectively, and are tuned empirically through experiments.

This loss function is designed to collaboratively optimize model performance under multimodal uncertainty conditions. The unimodal uncertainty loss simultaneously optimizes predicted values and variances to prevent overconfidence, while dynamically weighting modalities to emphasize those with higher confidence. The multimodal fusion loss directly supervises the accuracy of the final output, and the auxiliary loss promotes branch collaboration via intermediate supervision. The total loss balances multiple optimization objectives through weight allocation, enhancing model accuracy and robustness.

## 3. Experiments

### 3.1. Dataset and Preprocessing

In this study, we employ the publicly available SEED-VIG dataset [17], developed by the research team led by Baoliang Lü at Shanghai Jiao Tong University. The dataset was collected using a simulated driving system designed by the team to synchronously acquire electroencephalography (EEG) and electrooculography (EOG) signals with high-precision labeling. A total of 23 subjects participated in the experiment, during which EEG signals, EOG signals, and PERCLOS values were recorded simultaneously under fatigue-induced driving conditions.

**Figure 7:**
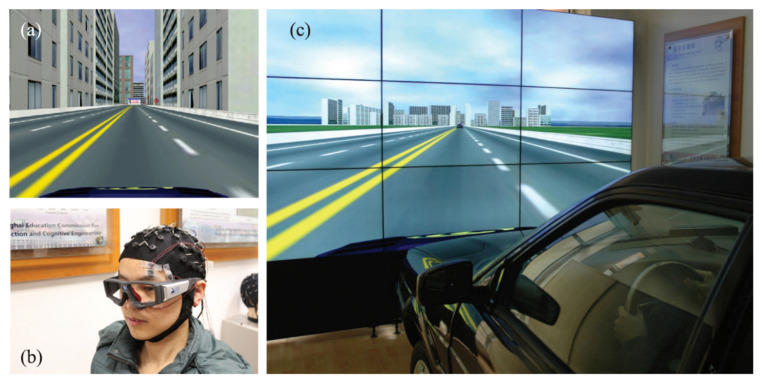
SEED-VIG dataset acquisition equipment and environment schematic: (**a**) Urban road scene constructed in virtual reality; (**b**) Neuroscan system for EEG and EOG signal acquisition with SMI eye-tracking glasses for gaze monitoring; and (**c**) Real vehicle equipped with basic driving controls.

As shown in Figure 7, data acquisition was conducted in a realistic vehicle cockpit equipped with a large liquid crystal display. The screen presented a simplified four-lane highway scenario, with unnecessary elements such as engine components removed to minimize distraction. Subjects controlled the virtual vehicle using a steering wheel and accelerator, with the driving scene updated in real time based on their operations. The road was designed to be predominantly straight and monotonous to effectively induce driver fatigue. Each subject performed a continuous driving task for approximately 2 h to ensure the collection of high-quality physiological signals sufficient to induce fatigue.

**Figure 8:**
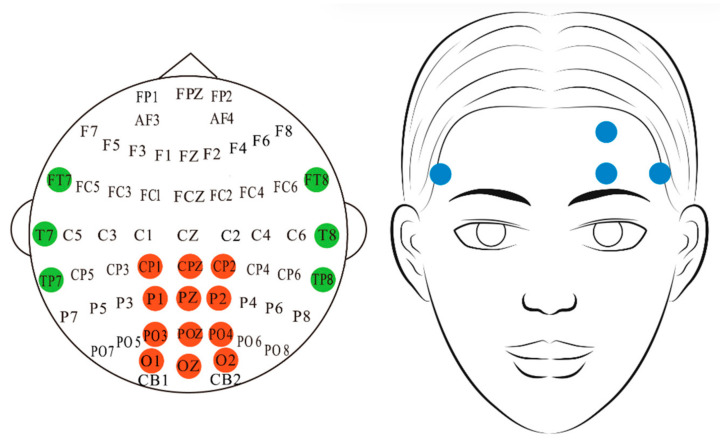
The electrode placement for EEG and EOG signals in the SEED-VIG dataset follows standard biosignal acquisition protocols.

As shown in Figure 8, the dataset includes EEG signals sampled at 200 Hz through 17 channels (following the international 10–20 system), as well as EOG signals captured from the forehead region. The EEG data provide two types of frequency-domain representations: full-frequency band features (1–50 Hz with 2 Hz resolution) and five sub-band features (Delta, Theta, Alpha, Beta, and Gamma bands), both including power spectral density (PSD) and differential entropy (DE). For the EOG signals, vertical and horizontal eye movement features were extracted using independent component analysis (ICA) and subtraction separation techniques. All data were annotated with fatigue levels based on the PERCLOS index (percentage of eyelid closure over the pupil per minute), which ranges from [0,1] and effectively reflects continuous changes in the subjects’ fatigue state.

In the data preprocessing stage, this study first performed systematic dimension adjustment and standardization on the raw physiological signals. Considering the characteristics of multimodal data, we placed the time-step dimension of EEG and EOG signals at the front and flattened the EEG frequency-domain features.

Eventually, three standardized data formats were formed: full-band EEG features (dimension: 20,355 × 4 × 425), EOG features (dimension: 20,355 × 3 × 36), and five-band EEG features (dimension: 20,355 × 4 × 85). To eliminate dimensional differences between channels and frequency bands, a channel-level Z-score standardization method was adopted, with the calculation formula given as follows: ${\hat{X}}_{B,C,F}=\frac{X_{B,C,F}-\mu_{C,F}}{\sigma_{C,F}+\epsilon }$, where $\mu_{C,F}$ and $\sigma_{C,F}$ represent the mean and standard deviation of each channel-frequency band, and $\epsilon =10^{-8}$ is used to ensure numerical stability.

In terms of experimental design, this study employs leave-one-out cross-validation. In each experiment, 885 samples from one subject are used as the test set, and the remaining 20,355 samples from the other 22 subjects serve as the training set. This approach is used to assess the model’s generalization ability to new subjects.

### 3.2. Parameter

In terms of experimental environment configuration, the hardware platform utilized an NVIDIA GeForce RTX 4090 GPU (equipped with 16,384 CUDA cores, 24 GB GDDR6X memory, and a 384-bit memory interface width), paired with a 20-core CPU and 80 GB RAM. The implementation was conducted on a Windows 10 Professional operating system using Python 3.6.13 and the PyTorch 1.10.2 framework. To assess the practical applicability of TMU-Net for real-time deployment, we evaluated its computational efficiency on this platform. The model demonstrated a low complexity of 0.003 GFLOPs. The average inference time for a single forward pass was 6.053 ms. When performing uncertainty quantification with 10 Monte Carlo samples, the average inference time was 9.408 ms, which is well below the 33.33 ms requirement for real-time processing at 30 FPS, confirming its strong potential for real-world deployment.

The unimodal feature extraction module adopts a hierarchical convolutional architecture to process multi-source physiological signals. For the three input modalities—EEG_2Hz (4 channels × 425 time-series points), EEG_5Bands (4 channels × 85 frequency-band features), and EOG (3 channels × 36 eye-movement features), they are first processed through a two-layer one-dimensional causal convolutional network. The convolution kernel size is set to 3, and a replication padding strategy is adopted (padding = 1). The first convolutional layer uniformly maps each modality to 16 channels, while the second layer performs differentiated processing: EEG signals are expanded to 64 channels, and EOG signals to 32 channels. To enhance the feature expression capability, a 4-head sparse convolutional attention mechanism (ConvSparseAttention) is introduced into the network. For temporal modeling, a 2-layer Transformer encoder is employed. The Transformer architecture for all modalities used 4 attention heads ($n_{h}$ = 4). For EEG signals, the model dimension ($d_{model}$) was set to 64, resulting in a key/query/value dimension of 16. For EOG signals, $d_{model}$ was set to 32, resulting in a key/query/value dimension of 8. The dimension of the feed-forward network within the Transformer was 128. All residual connections integrate LayerNorm and Dropout regularization (dropout rate *p* = 0.3).

In the multimodal fusion stage, features from each modality are first linearly projected into a 64-dimensional shared space for alignment. A three-head cross-modal attention mechanism (with a dropout rate of 0.1) is employed to establish dynamic inter-modal relationships. To achieve adaptive feature fusion, a two-layer gated MLP structure is designed, with both hidden layer dimensions of 64 and employing the Sigmoid activation function, combined with a variance-based weighting strategy (with a regularization coefficient λ = 0.001), incorporated to balance the contribution of each modality. Throughout the fusion process, residual connections are maintained, and LayerNorm is applied after each sub-module to stabilize the training procedure.

For model training, we employ the AdamW optimizer with differentiated learning rates for distinct modules: 4 × 10^−4^ for the EEG_2Hz module, 7 × 10^−4^ for the EEG_5Bands module, 3 × 10^−4^ for the EOG module, and 5 × 10^−4^ for the fusion module. The training batch size is fixed at 128, and training is conducted for 50 epochs. During inference, to evaluate model uncertainty, we performed 10 Monte Carlo sampling iterations (mc_samples = 10) while maintaining the dropout rate at 0.3. The final prediction was obtained by averaging the outputs of these 10 stochastic forward passes. The predictive uncertainty for each sample was quantified as the variance of these 10 outputs.

### 3.3. Experimental Results

#### 3.3.1. Fatigue Detection Performance

As shown in Table 1, the model achieved an average RMSE of 0.150599 and an average MAE of 0.123802 on the SEED-VIG dataset, demonstrating high prediction accuracy. Subject 10 exhibited the lowest RMSE, reaching 0.046472, and the lowest MAE, reaching 0.033936, indicating that the model predicted fatigue states for this individual with exceptional accuracy. In contrast, Subject 13 recorded the highest RMSE at 0.361604 and the highest MAE at 0.330456, suggesting that the model’s generalization capability is limited for certain individuals. The relatively large prediction errors for these two subjects are closely related to the quality of the participants and their physiological characteristics. Some physiological signals contained artifacts, and the noise patterns varied across individuals, ultimately leading to increased prediction errors. The standard deviations of RMSE and MAE, which are 0.067982 and 0.063950, respectively, reflect the model’s stability across different subjects and further highlight the variability in performance between individuals.

#### 3.3.2. Performance Comparison

As shown in Table 2, the proposed TMU-Net outperforms all baseline methods, achieving the best average RMSE of 0.1506 with a standard deviation of 0.0680. Compared to the best baseline DResNet, TMU-Net reduces RMSE by 4.01 percent and standard deviation by 7.48 percent, indicating improved accuracy and stability. This performance gain is attributed to its novel multimodal fusion architecture. TMU-Net leverages cross-modal attention to integrate complementary features from EEG_2Hz, EOG, and EEG_5Bands, enhancing sensitivity to fatigue changes. The uncertainty-based dynamic weighting improves robustness, while the ConvSparseAttention module significantly reduces computational cost without compromising key spatiotemporal feature extraction. Additionally, the Transformer-based unimodal encoder enables efficient global feature aggregation. These components work together to make TMU-Net highly effective for PERCLOS prediction, offering both high precision and strong robustness to individual variability and noise—critical for real-time driver fatigue monitoring.

#### 3.3.3. Comparison Between Unimodal and Multimodal Approaches

As shown in Table 3, the single-modal methods for fatigue detection are based on EEG_2Hz, EOG, and EEG_5Bands. Among the three single-modal experiments, the EOG modality achieved the best performance, with an average RMSE of 0.158179 and MAE of 0.122523, and standard deviations of 0.082630 and 0.070814, respectively, indicating higher stability and accuracy in predicting PERCLOS values. This superior performance may be attributed to EOG data being directly derived from eye movement tracking, which is highly correlated with PERCLOS labels based on eyelid closure duration, thus effectively capturing fatigue-related ocular movement features. However, EOG performed poorly for certain subjects, such as subject 13 (RMSE: 0.445883, MAE: 0.385439), possibly due to the influence of lighting conditions or device calibration issues. The EEG_2Hz modality yielded an average RMSE of 0.226376 and MAE of 0.184766, with standard deviations of 0.106922 and 0.098614, respectively. EEG_2Hz provides high-resolution frequency features that can capture fine-grained variations in brain electrical activity. Nevertheless, its performance exhibited substantial inter-subject variability, such as subject 12 with a high RMSE of 0.593778 and MAE of 0.532934, which may be related to the susceptibility of EEG signals to noise or individual differences in brain activity patterns. The EEG_5Bands modality recorded the highest average RMSE (0.267976) and MAE (0.224698), with standard deviations of 0.131726 and 0.119818, respectively, indicating the lowest prediction accuracy and stability among single-modal methods. Although EEG_5Bands reduces feature dimensionality by utilizing five frequency bands, this may lead to the loss of critical frequency details, impairing the model’s ability to capture complex fatigue-related EEG patterns.

Dual-modal fusion methods, by combining features from two modalities, outperform their respective single-modal counterparts, demonstrating the advantage of complementary feature integration. According to Table 3, compared to single-modal EEG_2Hz (RMSE: 0.226376, MAE: 0.184766), the EEG_2Hz+EOG combination reduces RMSE and MAE by 31.73% and 32.52%, respectively, while EEG_2Hz+EEG_5Bands reduces RMSE and MAE by 1.96% and 1.84%. Compared to single-modal EEG_5Bands (RMSE: 0.267976, MAE: 0.224698), the EOG+EEG_5Bands combination reduces RMSE and MAE by 41.73% and 43.88%, respectively, while EEG_2Hz+EEG_5Bands reduces RMSE and MAE by 17.17% and 16.26%. Compared to single-modal EOG (RMSE: 0.158179, MAE: 0.122523), EEG_2Hz+EOG and EOG+EEG_5Bands reduce RMSE by 2.27% and 1.26%, respectively, while MAE increases slightly by 1.75% and 2.95%. However, the MAE standard deviations for EEG_2Hz+EOG (0.066955) and EOG+EEG_5Bands (0.068813) are lower than that of single-modal EOG (0.070814), indicating significantly improved prediction consistency. The advantage of dual-modal methods lies in integrating complementary information from brain electrical activity and eye movement signals. For instance, EEG_2Hz+EOG effectively mitigates EEG_2Hz’s sensitivity to noise and EOG’s dependence on external conditions, while EOG+EEG_5Bands leverages EOG’s high correlation to enhance EEG_5Bands’ predictive capability. Although EEG_2Hz+EEG_5Bands shows smaller improvements, it still enhances performance by combining two EEG feature sets. The lower standard deviations of dual-modal methods compared to single-modal methods indicate reduced prediction variability, which is critical for fatigue detection applications across diverse subjects.

The multimodal fusion approach, integrating features from EEG_2Hz, EOG, and EEG_5Bands via the MultiModalFusion module with a cross-modal attention mechanism and uncertainty modeling through Monte Carlo Dropout and epistemic and aleatoric uncertainty estimation, achieves the best overall performance, with an average RMSE of 0.150599, MAE of 0.123802, and standard deviations of 0.067982 and 0.063950. Compared to the best single-modal EOG, RMSE is reduced by 4.78%, while MAE increases slightly by 1.05%, but the MAE standard deviation decreases from 0.070814 to 0.063950. Compared to the best dual-modal EEG_2Hz+EOG, RMSE and MAE are reduced by 2.58% and 0.74%, respectively, with the MAE standard deviation further reduced to 0.063950. Compared to EEG_2Hz and EEG_5Bands, RMSE is reduced by 33.46% and 43.79%, and MAE by 33.00% and 44.92%, respectively. The low standard deviations of the multimodal approach indicate the highest prediction stability, effectively mitigating limitations of single- and dual-modal methods through comprehensive feature integration, demonstrating superior adaptability to diverse physiological and behavioral characteristics across subjects.

#### 3.3.4. Temporal Prediction Results

**Figure 9:**
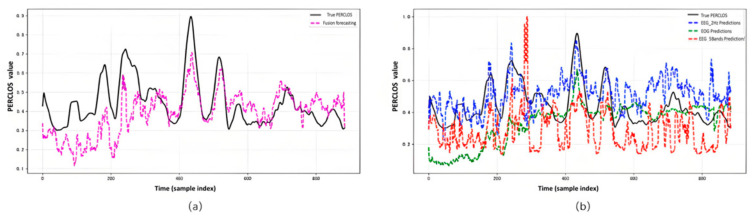
Comparison Between Predicted and True PERCLOS Over Time (Fold 23): (**a**) Multimodal fusion prediction; and (**b**) Individual Predictions of Unimodal EEG_2Hz, EOG, and EEG_5Bands.

Figure 9 presents a comparative analysis of multimodal and three unimodal prediction curves against the ground truth curve for the PERCLOS values of Subject 23. In Figure 9a, the multimodal fusion prediction curve exhibits a high degree of alignment with the ground truth in both trend and amplitude, particularly during fatigue peaks (sample points 200–600, PERCLOS > 0.6) and low-fatigue phases (sample points 400–800, PERCLOS < 0.2). This advantage stems from the MultiModalFusion module, which effectively integrates complementary information from EEG_2Hz, EOG, and EEG_5Bands through a cross-modal attention mechanism. Notably, the multimodal approach demonstrates greater sensitivity in responding to rapid changes in fatigue states, attributed to the robustness of uncertainty modeling in handling noise and dynamic variations. Although some deviations persist in certain peak and trough regions (e.g., an evident underestimation of the peak near sample point 450), the error remains relatively small compared to other model predictions. Figure 9b compares the prediction results of three unimodal models (EEG_2Hz: blue dashed line; EOG: green dashed line; EEG_5Bands: red dashed line). The EEG_2Hz prediction curve exhibits significant fluctuations and a tendency to overfit, closely aligning with some high-value points but deviating considerably elsewhere. The EOG curve is relatively stable but lacks sensitivity, underestimating many peaks. The EEG_5Bands curve, due to insufficient frequency band feature resolution, shows the largest fluctuations and severe oscillations with substantial noise, yielding poor overall fit despite occasional proximity to the ground truth. This experiment further confirms that the multimodal approach, with an RMSE of 0.150599 and an MAE of 0.123802, outperforms all unimodal models (EEG_2Hz: RMSE 0.226376; EOG: RMSE 0.158179; EEG_5Bands: RMSE 0.267976), highlighting its superior temporal resolution and prediction stability.

#### 3.3.5. Comparative Study on PERCLOS Prediction

As shown in Table 4, the ablation study results demonstrate that the complete TMU-Net model achieves optimal performance in PERCLOS prediction, with an RMSE of 0.151 ± 0.068 and an MAE of 0.124 ± 0.064, surpassing all ablated configurations and confirming the significant contribution of synergistic interactions among modules to enhanced prediction accuracy. The convolutional sparse attention module, by integrating local convolution and global attention mechanisms during unimodal feature extraction, effectively captures complex spatiotemporal patterns in EEG signals at 2 Hz and across five bands, as well as EOG signals, which are critical for PERCLOS prediction. Removal of this module results in a substantial performance decline, with an RMSE of 0.179 ± 0.126 and an MAE of 0.145 ± 0.112, exhibiting the highest mean and standard deviation, underscoring its pivotal role in both prediction accuracy and stability. The absence of this mechanism impairs the model’s ability to model complex signals, leading to significantly increased prediction errors and variability, particularly under noisy conditions. The cross-modal attention module enhances multimodal interactions, such as synergy between EEG brain activity and EOG eye movement features, improving fusion effectiveness. Its removal causes a modest performance drop, with an RMSE of 0.157 ± 0.073 and an MAE of 0.129 ± 0.069, but the decline is mitigated by other fusion mechanisms, with a slightly elevated standard deviation indicating its secondary contribution to stability. The multi-scale feature extraction module, by integrating multimodal predictions and uncertainty information, strengthens feature representation during the fusion stage. Its removal yields performance comparable to cross-modal attention, with an RMSE of 0.154 ± 0.067 and an MAE of 0.126 ± 0.062, with a lower standard deviation suggesting improved stability through smoother feature distributions. The uncertainty weight calculation module, via dynamic weighting based on modal uncertainty, prioritizes reliable modalities, such as high-quality EOG signals, enhancing fusion robustness. Its removal leads to a performance decline, with an RMSE of 0.155 ± 0.075 and an MAE of 0.127 ± 0.070, with a slightly higher standard deviation reflecting its auxiliary role in stability, particularly when signal quality varies. The complete model, integrating all modules, extracts high-quality features in the unimodal stage and achieves efficient fusion in the multimodal stage, fully leveraging the spatiotemporal information and complementarity of multimodal signals to attain the lowest error and moderate stability. The results highlight the convolutional sparse attention module as the most critical, followed by cross-modal attention, multi-scale feature extraction, and uncertainty weight calculation. The complete model’s superior RMSE and MAE demonstrate robust predictive performance, providing theoretical support for the TMU-Net design and guidance for model optimization in applications such as driver fatigue monitoring.

#### 3.3.6. Attention Mechanism Analysis

**Figure 10:**
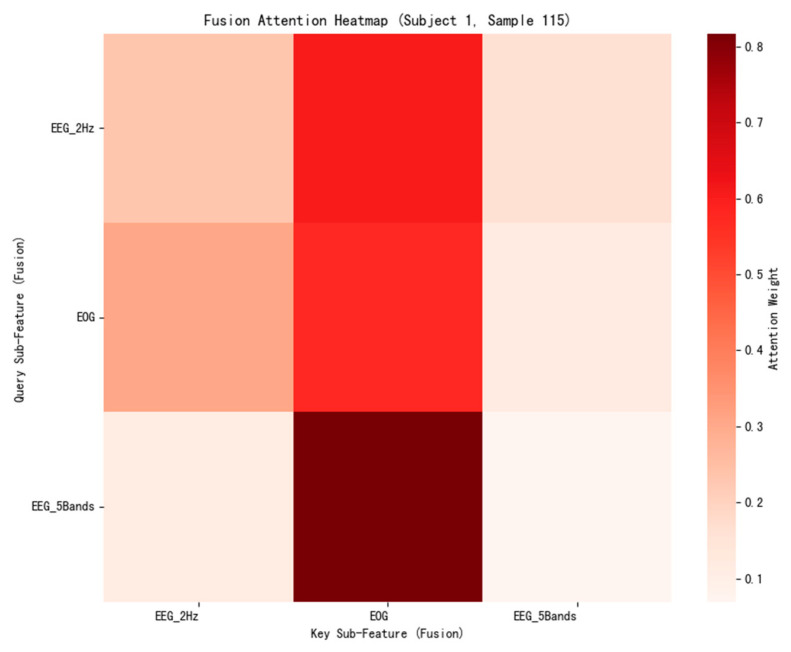
Fusion attention heatmap of Subject 1 (Sample 115).

As shown in Figure 10, the model exhibits distinct variations in the allocation of attention weights across different physiological signal submodalities. Taking Subject 1 (Sample 115) as an example, the PERCLOS value is approximately 0.29, approaching or reaching the threshold for moderate fatigue [31], indicating a significant fatigue state during this period. In this state, the model assigns higher attention weights to EOG and EEG_2Hz, while the attention to EEG_5Bands is notably lower. This distribution pattern is highly consistent with the physiological mechanisms of fatigue development: in the early stages of fatigue, behavioral changes (such as increased blink frequency and slowed eye movements) typically precede central nervous system responses, making eye movement signals more effective in capturing critical cues of initial fatigue. The higher attention weight assigned to EOG during this stage demonstrates that the model effectively recognizes its diagnostic value in fatigue monitoring. Meanwhile, EEG_2Hz, corresponding to low-frequency brain activity (primarily in the theta band), is associated with the brainstem–thalamus-cortex network and serves as an electrophysiological marker of early central nervous fatigue. The increase in its attention weight reflects fluctuations in brain regulatory capacity. In contrast, EEG_5Bands, which integrates features across multiple frequency bands, does not yet exhibit sufficient discriminative power in the early stages of fatigue progression, resulting in lower attention allocation by the model. This attention mechanism not only demonstrates the model’s strong task-focused capability in its architecture but also physiologically reflects the fatigue evolution process in a scientifically interpretable manner, providing a robust and explainable basis for fatigue detection based on multimodal signals.

## 4. Discussion

The experimental results demonstrate that TMU-Net achieves superior driver fatigue detection performance on the SEED-VIG dataset, with an average RMSE of 0.1506 ± 0.0680 and MAE of 0.1238 ± 0.0640, outperforming baseline methods such as DResNet (RMSE: 0.1569 ± 0.0735) by reducing RMSE by 4.01% and standard deviation by 7.48%. These improvements stem from TMU-Net’s multimodal fusion architecture, which integrates EEG (2Hz full-band and five-band features) and EOG signals through cross-modal attention, uncertainty-weighted gating mechanisms, and ConvSparseAttention modules. By leveraging uncertainty estimation to dynamically adjust the contribution of each modality, the model enhances robustness to noise and inter-subject variability, addressing key limitations in signal quality fluctuations and cross-subject generalization identified in prior studies. Shi et al. [32] proposed a multimodal fusion approach based on time-space-frequency features, utilizing a convolutional autoencoder (CAE) to integrate EEG and EOG signals, achieving an RMSE of 0.08 and a correlation coefficient of 0.96 on the SEED-VIG dataset, and thereby validating the potential of multimodal fusion to enhance detection accuracy.

The comparison between unimodal and multimodal approaches highlights the synergistic advantages of multimodal fusion. Using EOG alone yields a competitive RMSE of 0.1582 ± 0.0826, but its performance degrades under conditions such as low lighting or when subjects wear glasses. EEG-based modalities (2Hz and five-band) provide complementary neurological information but are limited by higher variability (RMSE of 0.2264 ± 0.1069 for EEG_2Hz and 0.2680 ± 0.1317 for EEG_5Bands), likely due to motion artifacts and inter-subject physiological differences [16,25]. Consistent with findings by Alghanim et al. [33], TMU-Net’s multimodal fusion captures interactions between eye movement (EOG) and brain activity (EEG) through cross-modal attention mechanisms, achieving reductions in RMSE and MAE of 4.82% and 0.55%, respectively, compared to the best unimodal approach (EOG), and reductions of 33.46% and 43.79% in RMSE and 33.00% and 44.92% in MAE compared to EEG_2Hz and EEG_5Bands, respectively.

The ablation study further elucidates the contributions of TMU-Net’s components. The ConvSparseAttention module is critical for unimodal feature extraction, and its removal leads to a significant performance decline, underscoring its pivotal role in capturing spatiotemporal dependencies in EEG and EOG signals. The cross-modal attention and uncertainty-weighted gating mechanisms enhance fusion by dynamically prioritizing reliable modalities, as confirmed by attention heatmap analysis. These mechanisms address the limitations of static-weighting schemes noted in prior studies, improving robustness to signal noise. TMU-Net’s prediction standard deviation (0.0680) is lower than that of baseline methods such as SVR (0.2076) and MLP (0.1822), indicating improved cross-subject generalization and partially mitigating the 30–40% performance degradation for new subjects reported in earlier research. Similarly, Nemcová et al. [34] demonstrated that a multimodal neural networks integrating EEG, EOG, and facial image features via a coupling mechanism achieved over 95% classification accuracy on the DROZY dataset, further validating the efficacy of dynamic feature fusion in enhancing generalization.

Despite these advancements, TMU-Net exhibits certain limitations. The model shows considerable performance variability across subjects, with Subject 13 yielding an RMSE of 0.3616 and MAE of 0.3305, likely due to signal artifacts or physiological differences. Although uncertainty modeling mitigates some noise effects, its efficacy is limited in cases of severe signal degradation. Furthermore, the use of the SEED-VIG dataset, collected in a virtual reality environment, introduces constraints. The potential for simulator sickness among participants [35] could contaminate physiological signals (e.g., causing atypical eye movements or brain activity), thereby confounding fatigue assessment, and this factor was not monitored or controlled for in the original dataset. Several practical barriers remain for real-world deployment. Sensor discomfort from prolonged wear of EEG/EOG devices may hinder user adoption, necessitating research into dry-electrode or non-contact alternatives. Finally, the high cost and complexity of integrating multi-modal physiological sensors present scalability challenges.

Future research can explore the following directions: First, incorporating adversarial domain adaptation or transfer learning could further reduce the 30–40% performance gap across subjects by leveraging graph convolutional networks to enhance the cross-subject generalization of EEG signals. Second, optimizing TMU-Net’s computational efficiency through model pruning or lightweight attention mechanisms would facilitate real-time applications. Third, validating the model on diverse real-world driving datasets, such as the DROZY dataset used by Nemcová et al., which includes multimodal data under real sleep deprivation conditions, would strengthen its practical applicability. Future data collection efforts should also incorporate screening tools like the Simulator Sickness Questionnaire (SSQ) to identify and mitigate the impact of simulator sickness on data quality. Finally, integrating additional modalities, such as heart rate variability or vehicle dynamics parameters, could further improve detection accuracy by capturing a broader range of fatigue indicators. In conclusion, TMU-Net significantly advances driver fatigue detection through multimodal fusion, uncertainty quantification, and Transformer-based feature extraction. Its superior accuracy and robustness highlight its substantial potential for enhancing road safety via reliable real-time fatigue monitoring. Addressing the aforementioned limitations will further enhance its applicability in real-world driving scenarios.

## 5. Conclusions

This study proposes a novel driver fatigue detection framework, TMU-Net, which integrates EEG (2Hz full-band and five-band features) and EOG signals, combining cross-modal attention, uncertainty-weighted gating mechanisms, and a ConvSparseAttention module to significantly enhance the accuracy and robustness of fatigue detection. Compared to single-modal approaches, multi-modal fusion captures complementary information from EEG and EOG signals, substantially reducing prediction errors and improving robustness to noise and inter-subject variability through uncertainty modeling.

The core contribution of TMU-Net lies in its innovative architectural design. The ConvSparseAttention module effectively captures spatiotemporal features of physiological signals, the cross-modal attention mechanism integrates multi-modal information, and the uncertainty-weighted gating mechanism dynamically adjusts modal contributions to enhance adaptability to complex driving scenarios. Attention heatmap analysis further reveals the model’s prioritized focus on EOG and low-frequency EEG signals, which aligns closely with the physiological mechanisms of early fatigue. These features enable TMU-Net to demonstrate superior performance and stability in PERCLOS prediction tasks, offering a reliable solution for real-time driver fatigue monitoring with significant potential for road safety applications.

However, TMU-Net has room for improvement. Inter-subject performance variability (e.g., an RMSE of 0.3616 for Subject 13) indicates that the model’s robustness to extreme signal artifacts requires further enhancement, and its computational complexity limits real-time deployment in resource-constrained environments. Future research could improve cross-subject generalization by incorporating adversarial domain adaptation, optimizing the model architecture to reduce computational costs, and validating performance on real-world driving datasets. Additionally, integrating supplementary modalities, such as heart rate variability or vehicle dynamics parameters, could further enhance detection accuracy. Subsequent studies will focus on addressing these limitations to improve temporal coherence and classification accuracy.

## Figures and Tables

**Table 1:** Performance evaluation using LOOCV.

Index	RMSE	MAE
1	0.118939	0.096812
2	0.110041	0.089023
3	0.230304	0.191571
4	0.169818	0.143578
5	0.101254	0.076963
6	0.128247	0.098166
7	0.116746	0.087638
8	0.161194	0.11413
9	0.097768	0.076226
10	0.046472	0.033936
11	0.194315	0.165127
12	0.255315	0.222368
13	0.361604	0.330456
14	0.157339	0.103876
15	0.066419	0.048681
16	0.149974	0.126567
17	0.198523	0.168296
18	0.138114	0.121762
19	0.073681	0.05776
20	0.140626	0.112973
21	0.135539	0.121048
22	0.18023	0.159634
23	0.131308	0.100847
Avg	0.150599	0.123802
Std	0.067982	0.06395

**Table 2:** Performance comparison with previous methods on SEED_VIG.

Methods	RMSE
SVR	0.2071 ± 0.2076
MLP	0.1942 ± 0.1822
VIGNet [26]	0.1876 ± 0.1903
DG-DANN [27]	0.1543 ± 0.1490
MFSAN [28]	0.1780 ± 0.1559
MART (w/o Domain Generalization) [29]	0.1556 ± 0.1311
DResNet [30]	0.1569 ± 0.0735
TMU-Net	**0.1506 ± 0.0680**

**Table 3:** Performance comparison of unimodal versus multimodal fusion approaches on SEED_VIG.

Modality	RMSE	MAE
EEG_2HZ	0.226376 ± 0.106922	0.184766 ± 0.098614
EOG	0.158179 ± 0.08263	**0.122523** ± 0.070814
EEG_5BANDS	0.267976 ± 0.131726	0.224698 ± 0.119818
EEG_2HZ+EOG	0.154578 ± 0.072935	0.124668 ± 0.066955
EEG_2HZ+ EEG_5BANDS	0.221911 ± 0.089878	0.188159 ± 0.083861
EOG+ EEG_5BANDS	0.156201 ± 0.070616	0.126112 ± 0.068813
All Modalities	**0.150599 ± 0.067982**	0.123802 ± **0.063950**

**Table 4:** Performance impact of removing key components in TMU-Net.

Model Configuration	RMSE	MAE
No_ Cross-Modal Attention	0.157 ± 0.073	0.129 ± 0.069
No_ ConvSparseAttention	0.179 ± 0.126	0.145 ± 0.112
No_ Multi-Scale Feature Extraction	0.154 ± 0.067	0.126 ± 0.062
No_ Uncertainty Weight Calculation	0.155 ± 0.075	0.127 ± 0.070
Full Mode	0.151 ± 0.068	0.124 ± 0.064

## Data Availability

The original data presented in the study are openly available at https://bcmi.sjtu.edu.cn/home/seed/index.html, accessed on 27 August 2025.
